# Traffic-related pollution history (1994–2014) determined using urban lake sediments from Nanjing, China

**DOI:** 10.1371/journal.pone.0255395

**Published:** 2021-08-02

**Authors:** Gengyu Liu

**Affiliations:** 1 School of Geographical Sciences, Fujian Normal University, Fuzhou, China; 2 Fuzhou Investigation and Surveying Institute, Fuzhou, China; Institute for Advanced Sustainability Studies, GERMANY

## Abstract

With the development of urbanisation and the increasing number of modern vehicles, traffic contamination has become an important source of environmental pollution. Most previous studies have focused on using roadside soil or plants to determine the spatial pattern of traffic pollutants along roads and the factors that influence this pattern, whereas few studies have reconstructed pollution histories caused by traffic using suitable methods. In this study, two gravity cores were obtained from Qianhu Lake, which is in the Zhongshan tourist area of Nanjing City and is distant from industrial areas. An accurate chronological framework covering the period from 1994 to 2014 was established using the correlation between the variation in grain size of the sediment cores and the variation in annual rainfall in Nanjing City. Moreover, magnetic and chemical parameters were also measured, and the results demonstrated that concentration-related magnetic parameters exhibited different correlations with different heavy metal concentrations. These correlations were significantly positive for *Zn*, *Pb*, and *Co*; weakly positive for Ni; absent for Cr; and negative for V. Combined with statistical data on industrial emissions and private cars in Nanjing City since 1994, the observed variations in magnetic susceptibility, anhysteretic remanent magnetisation, saturation isothermal remanent magnetisation, *Zn*, *Pb*, and *Co*, were controlled by traffic activities in the tourist area but not by industry. Therefore, the variations in these parameters record the traffic pollution history of the study area. Combined with the obtained chronological framework, the traffic-related pollution history could be divided into two stages: 1) from 1994 to 2003, when traffic-related pollution became increasingly serious because of the exponential increase in the number of private cars and the prosperity of tourism; 2) from 2003 to 2014, when traffic-related pollution continuously increased but at a much slower rate than in stage 1. This slower rate of increase was probably related to the maximum carrying capacity of the tourist area and technological innovations in automobile manufacturing, as well as improvements in fuels.

## Introduction

With the development of urbanisation and industrialisation, the number of modern vehicles and the total length of highways have increased rapidly. In China, the number of cars reached more than 154 million, and the total length of highways rose to >100,000 km by late 2014 [1]; thus, traffic contamination has become an important source of environmental pollution. Motorised vehicles release large amounts of particulate matter (PM) and other pollutants into the atmosphere via fuel combustion, as well as abrasion of tyres, brake linings, and road surfaces [2–4]. This pollution has increased the frequency of numerous acute and chronic diseases [5–9]. Previous studies have shown that traffic activities generate large amounts of carbon and nitrogen oxides, heavy metal contamination, and magnetic phases [4, 10–15]. Moreover, there are significant positive correlations between heavy metal concentrations and magnetic parameters, and magnetic parameters can be used to monitor the source, distribution, and extent of heavy metal pollution [4, 14, 16–20]. Thus, an increasing number of studies have combined magnetic and chemical parameters to investigate traffic-related pollution. In general, roadsides are the most seriously contaminated areas, and most previous studies have focused on using roadside soil or plants to determine the spatial pattern of traffic pollutants along roads and the factors that influence this pattern [10, 14, 16, 17, 21–26]. Although biological targets (e.g. tree leaves) are easily available with high spatial resolution, they can only record contamination over short periods and cannot reveal the contamination history. In contrast, soil can accumulate environmental pollutants for many years; however, soil only provides a time-averaged value of the degree of pollution without accurate chronological information. Hence, previous studies have only provided qualitative descriptions of pollution that accumulated within a certain period of time (a few days to several years), and there is a lack of historical reconstructions of traffic pollution using suitable methods. Such historical reconstructions are valuable for assessing present and potential future risks associated with pollutants stored in sediment [27].

A prerequisite for reconstructing pollution history is that the records should be continuous and contain no disturbances. Moreover, they should contain accurate chronological information. Lake sediments that deposited continuously can be dated, thus offering a unique opportunity for studying pollution history. For example, Hu et al. (2003) [28] reconstructed the acidification process of sediments from Lake Yangzonghai in southwest China using magnetic parameters and heavy metals in combination with ^137^Cs and ^210^Pb dating methods. Yang et al. (2009) [29] found that the magnetic properties of lake sediments provided an excellent record of historical industrial activity and other anthropogenic disturbances in an urban lake catchment. Ma et al. (2015) [30] studied the magnetic and chemical parameters of sediments from a reservoir in Linfen, China, and quantitatively reconstructed the atmospheric pollution history in this region since 1979. Kang et al. (2016) [31] collected eight lake sediment cores and one glacier ice core from the Himalaya–Tibet region and reconstructed the deposition history of atmospheric Hg over the past 500 years at high resolution. Therefore, lake sediments can accurately record anthropogenic pollution conditions.

However, most previous studies on pollution history reconstruction have assessed all anthropogenic activities, primarily industrial activities, while few studies have disentangled traffic pollution from the overall signal or reconstructed pollution history related to traffic activity. In this study, two gravity cores were obtained from Qianhu Lake, which is in the Zhongshan tourist area of Nanjing City, and detailed magnetic and chemical parameters were measured. The main aims of this study are to 1) establish an accurate chronological framework for the core sediments, 2) identify the sources of magnetic particles and heavy metals, and 3) reconstruct the history of traffic rather than Industrial pollution in the study area.

## Materials and methods

### Ethics

No specific permits were required for the described filed studies.

### Materials

Qianhu Lake (latitude 32°259” N, longitude 118°4931” E) is located in the Zhong Mountain tourist area of Nanjing City in Jiangsu Province, China. It is far away from industrial areas and experiences an annual rainfall of approximately 1363 mm and an annual mean temperature of 15.6°C. No rivers flow into the lake; thus, lake water is mainly derived from rainfall and catchment runoff. The vegetation cover around the lake is extensive. There are many scenic spots near Qianhu Lake, most of which are famous in China and have been open to the public since the 1990s. In addition, a large parking area and conservatory area were built in the north-western part of the lake shore. Therefore, the most significant anthropogenic activity affecting the study area is tourism. In May 2015, two sediment cores (QH1 and QH2) were obtained using gravity samplers at different locations in the centre of the lake. The total length of core QH1 was 31 cm, and core QH2 was 39 cm. Both cores were dominated by black mud without obvious lithological changes. They were sub-sampled at 1 cm intervals and then dried naturally indoors.

To measure the magnetic parameters of the cores, we ground the air-dried samples and placed them in non-magnetic plastic boxes (8*cm*^3^). We measured the low-frequency magnetic susceptibility (*χ*_*lf*_), high-frequency magnetic susceptibility (*χ*_*hf*_), and anhysteretic remanent magnetisation (ARM) of all the samples. The *χ*_*lf*_ was measured using a Bartington MS2-B meter at a low frequency (470 Hz), and the *χ*_*hf*_ was measured at a high frequency (4700 Hz). The percentage frequency-dependent susceptibility (*χ*_*fd*_%) can be calculated by *χ*_*lf*_ and *χ*_*hf*_, and the formula is $\chi_{fd}\left(\%\right)=\left(\frac{\left(\chi_{lf}-\chi_{hf}\right)}{\chi_{lf}}\right)\times 100$. The ARM was measured using a Molspin Minispin magnetometer after magnetisation using a D-2000 alternating field demagnetiser. The peak alternating field (AF) was 100 mT, and the direct current (DC) bias field was 50*μT*. Based on previous experiments, we selected some samples to measure their other magnetic parameters, including isothermal remanent magnetisation (IRM) acquisition curves, IRM demagnetisation curves, high-temperature magnetic susceptibility curves (*κ*–*T* curves), and magnetic hysteresis loops. The IRM acquisition curves were acquired under progressively increasing magnetic fields, and the IRM demagnetisation curves were acquired under progressively decreasing magnetic fields using an IM-10–30 pulse magnetiser, and then measured using a Minispin magnetometer. Saturation isothermal remanent magnetisation (SIRM) is an IRM with an external field of 1 T. High-temperature *κ*–*T* curves were measured using an Agico KLY-3 Kappabridge and a CS-3 high-temperature furnace. Magnetic hysteresis loops were obtained using a variable field translation balance (VFTB) with a maximum field of 1000 mT.

An appropriate amount of each sample was ground carefully to <200 mesh and then treated with hydrofluoric acid (HF), nitric acid (*HNO*_3_), and hydrochloric acid (HCl). The contents of *Zn*, *Pb*, *Co*, *Cr*, *Ni*, and *V* were determined using an X-Series II inductively coupled plasma–mass spectrometer (ICP-MS).

Grain size was measured using a Mastersizer 2000 laser particle size analyser. All measurements were completed at the Key Laboratory for Subtropical Mountain Ecology at Fujian Normal University.

## Results

**Fig 1:**
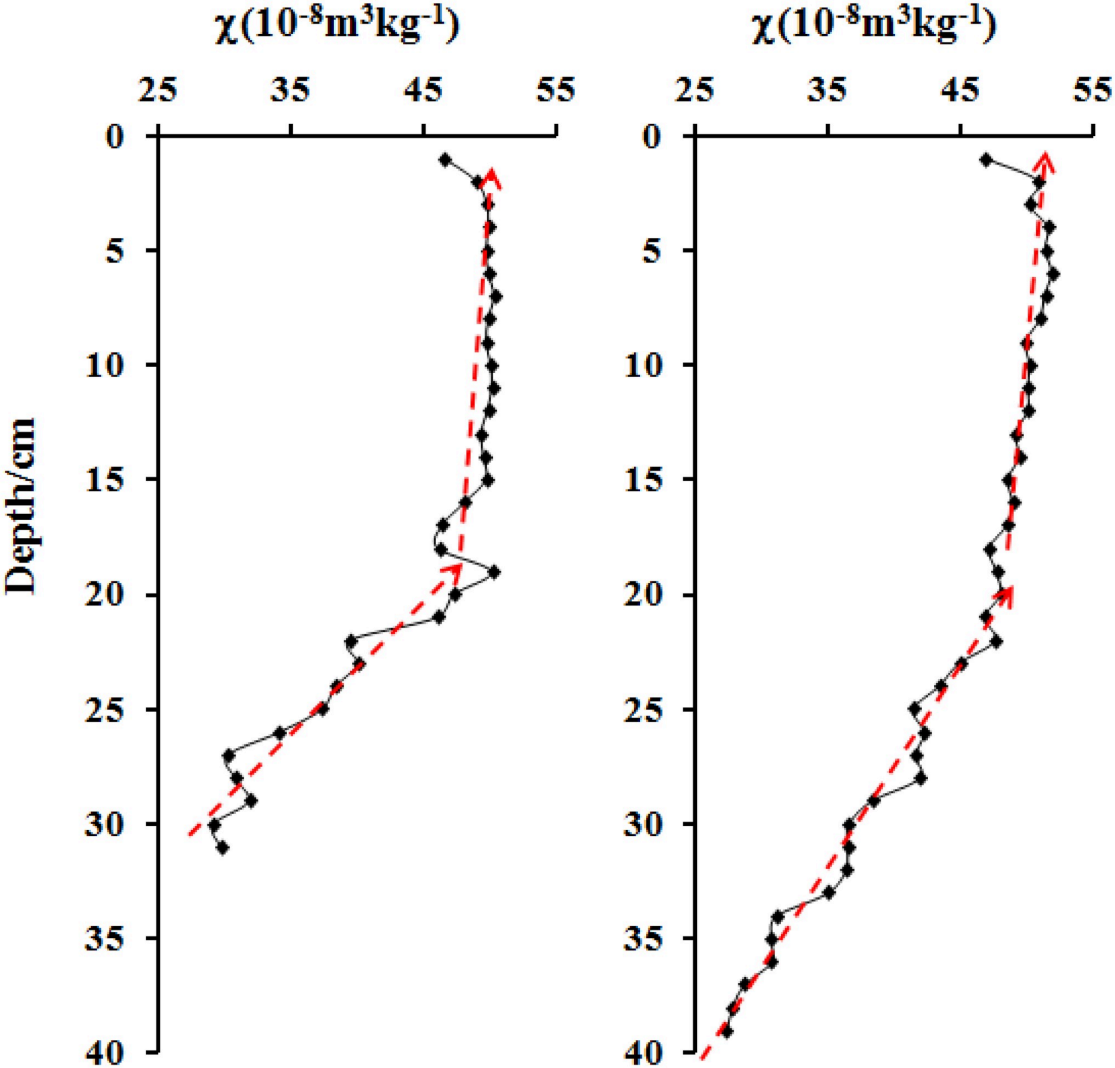
Magnetic susceptibility variation of cores QH1 (left) and QH2 (right).

Fig 1 shows the *χ* curves of cores QH1 and QH2. In core QH1, *χ* increased rapidly between 31 cm to 20 cm, and then increased slowly between 20 cm to the top. In core QH2, *χ* increased rapidly between 39 cm to 20 cm, and then increased slowly between 20 cm to the top. The *χ* curves of both cores were clearly very similar. As the two cores came from different locations in the centre of the lake, the consistency of their *χ* values suggests that they represent the overall magnetic properties of the lake sediments. Because core QH2 was 8 cm longer than QH1, QH2 was selected for detailed analysis.

### High-temperature *κ*–T curves

**Fig 2:**
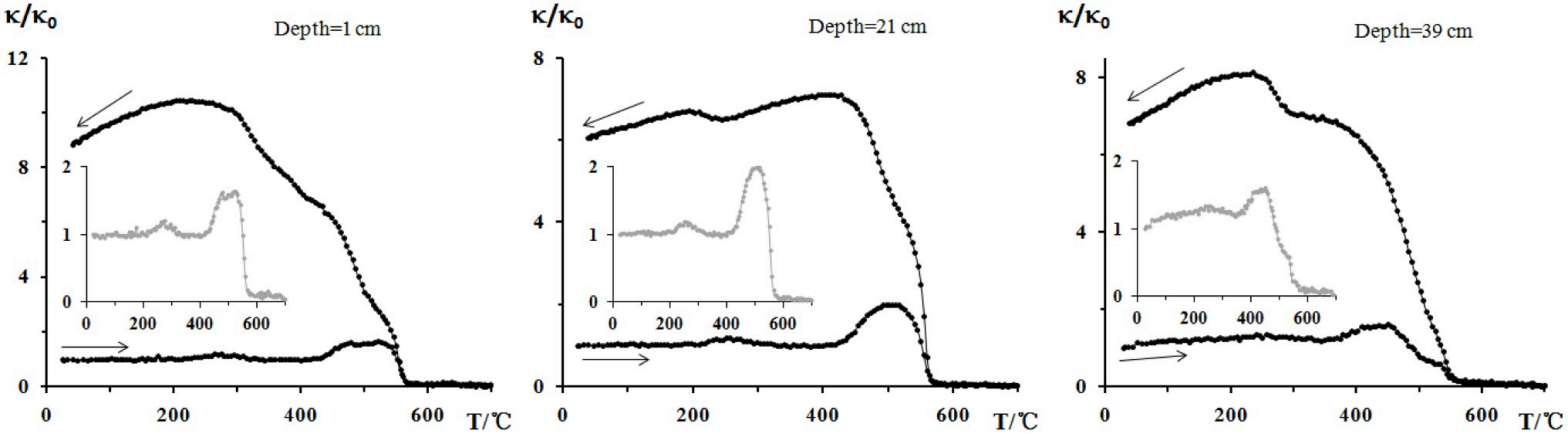
High-temperature *κ*–T curves of pilot samples (grey lines indicate heating curves).

The type of magnetic mineral can be identified by the Curie temperature (Tc) or the specified temperature during the heating process using the high-temperature *κ*–T curves. Fig 2 shows the high-temperature *κ*–T curves for three samples at different depths (1 cm, 21 cm, and 39 cm). The high-temperature *κ*–T curves of all three samples exhibited special changes at 250°C, 300–400°C, 500°C, and 580°C. Weak peaks were observed near 250°C in all *κ*–T curves, which may relate to the formation of new strongly magnetic minerals during the heating process. A significant reduction in the –T curves was observed at 300–400°C, which might relate to the instability of maghemite after heating. A pedogenic origin from the surrounding catchments could be the main source of this maghemite [32–36], which then converted to hematite by heating [37]. When the samples were heated to 500°C, the *κ*–T curves of all samples were enhanced, indicating the presence of some paramagnetic minerals or weak magnetic changes to strong ferro(i)magnetic phases [38–40]. In addition, a significant Tc of ∼580°C was observed in all samples, suggesting the existence of magnetite. In the sample from a depth of 39 cm, there was also an obvious Tc of ∼520°C, which may relate to titanic magnetite with a Tc value lower than that of pure magnetite [41]. By analysing high-temperature *κ*–T curves, signals of magnetite and maghemite could be observed in the samples, which are soft magnetic minerals.

### Magnetic hysteresis loops, IRM acquisition, and demagnetisation curves

**Fig 3:**
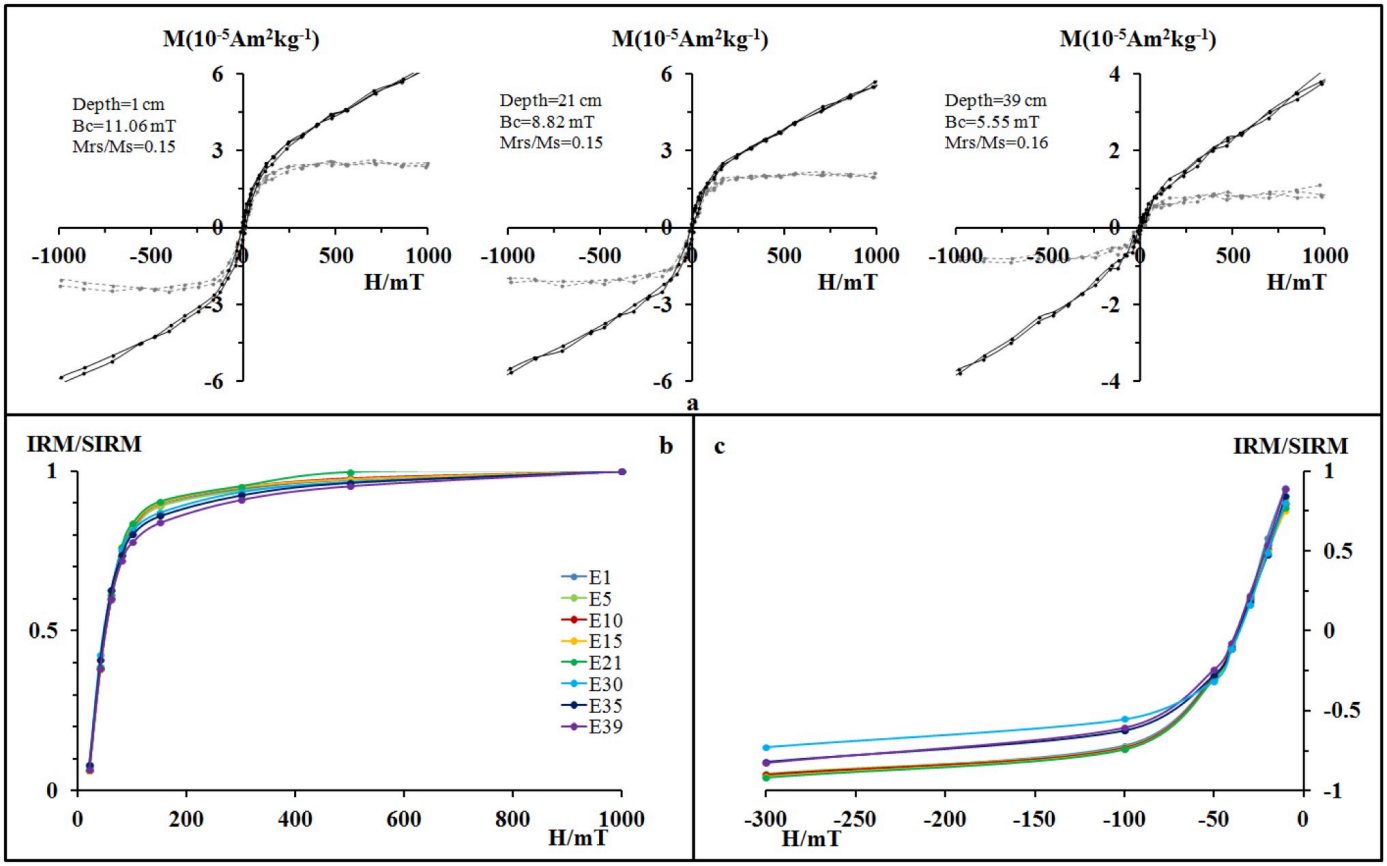
Magnetic hysteresis loops (a) (the black lines indicate the original curves, and the grey lines indicate the slope-corrected curves), IRM acquisition curves (b), and backfield curves (c) of pilot samples. The numbers after E indicate the depth in centimetres.

The coercivities of magnetic minerals are related to the type of magnetic minerals, the iron oxide concentration, and the particle size. The coercivities of magnetic minerals can be obtained from magnetic hysteresis loops [42]. As shown in Fig 3a, the Bc of the three samples varied from 5.55 mT to 11.06 mT, and the magnetic hysteresis loops after paramagnetism correction were closed. In addition, magnetisation was nearly saturated in an extra magnetic field of 300 mT, which is a typical characteristic of low coercivity. The main magnetic minerals in these samples were soft magnetic minerals (e.g. magnetite and maghemite). When the uncorrected initial curves exceeded 300 mT, the magnetisation increased with increasing magnetic field intensity, indicating the existence of paramagnetic or hard magnetic phases.

Generally speaking, IRM does not contribute to (super) paramagnetic minerals, and at an extra magnetic field of 300 mT, soft magnetic minerals (e.g. magnetite and maghemite) will saturate. In contrast, hard magnetic minerals (e.g. hematite and goethite) are far from saturated even at 1 T of extra magnetic field. Therefore, the relative proportions of soft magnetic minerals and hard magnetic minerals can be roughly estimated from the IRM-obtained curves. As shown in Fig 3b, the curves of all samples showed an obvious saturation trend at a field of 1 T, and under the 300 mT external magnetic field, the IRM of all selected samples occupied 91%–95% of their SIRM (at 1 T). Moreover, Fig 3c shows that the remanent magnetisation could be easily demagnetised at a reverse magnetic field of 30–40 mT. This conclusion is consistent with the high-temperature T curves (Fig 2) and hysteresis loops (Fig 3a), whereby the main magnetic minerals in all samples were soft magnetic minerals such as magnetite and maghemite.

### Vertical variations in magnetic parameters and heavy metal concentrations

**Fig 4:**
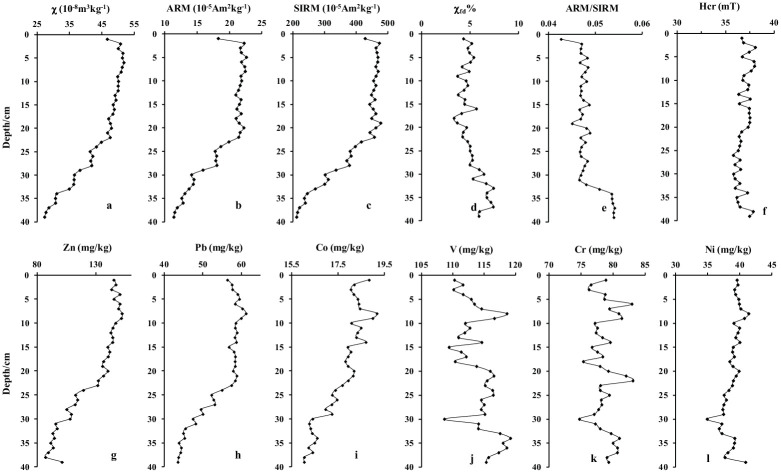
Vertical variations in magnetic parameters (a-f) and heavy metal concentrations (g-l) of core QH1.

In general, the magnetic mineral types, concentrations, and partial size distribution determine the *χ*, *ARM*, and *SIRM* of a sample. If the main magnetic minerals in a sample are soft magnetite (e.g. magnetite or maghemite), *ARM* can be used to indicate the number of fine magnetite particles (especially single domain (SD) magnetite particles; [42]). Fig 4a–4c show that the trends of *χ*, *ARM*, and *SIRM* were similar. The process of change of *χ*, *ARM*, and *SIRM* could be divided into three stages: 1) they increased rapidly from the bottom to a depth of 20 cm; 2), they increased slowly from 20 cm to 2 cm; and 3) they gradually decreased near the top. This tendency indicates that the samples were controlled by soft magnetic minerals such as magnetite and maghemite. The content of superparamagnetic (SP) magnetite particles can be expressed as *χ*_*fd*_%, and the presence of SD magnetite particles in the samples can be indicated by the *ARM*/*SIRM* ratio. As shown in Fig 4d, the trend of fd% could be divided into two stages: 1) *χ*_*fd*_% decreased continuously from the bottom to 20 cm; and 2) *χ*_*fd*_% fluctuated steadily from 20 cm to the top. Fig 4e shows that *ARM*/*SIRM* ratio had four stages: 1) it was stable from the bottom to 34 cm; 2) it decreased rapidly from 34 cm to 31 cm; 3) it fluctuated near a relatively constant value from 20 cm to 2 cm; and 4) it decreased significantly from 2 cm to the top. The ratios of soft and hard magnetic minerals are indicated by Hcr. Fig 4f shows that the Hcr of all samples ranged from 35 mT to 40 mT, indicating that all samples were dominated by soft magnetic minerals such as magnetite and maghemite. As shown in Table 1, we analysed the correlation between the magnetic parameters and each heavy metal element. There were significant positive correlations between *ARM*, and *SIRM*, and the correlation coefficient between them was 0.99. However, these three magnetic parameters were negatively correlated with *χ*_*fd*_% (correlation coefficients (r values) of -0.79 to -0.83) and the *ARM*/*SIRM* ratio (r values of -0.69 to -0.78).

**Table 1:** Pearson’s correlation coefficients (r) between magnetic properties and heavy metal concentrations.

	*χ*	*ARM*	*SIRM*	*χ*_*fd*_%	*ARM*/*SIRM*	*Zn*	*Pb*	*Co*	*Ni*	*Cr*	*V*
*χ*	1										
*ARM*	0.99	1									
*SIRM*	0.99	0.99	1								
*χ*_*fd*_%	-0.79	-0.81	-0.83	1							
*ARM*/*SIRM*	-0.76	-0.69	-0.78	0.69	1						
*Zn*	0.96	0.95	0.95	-0.78	-0.66	1					
*Pb*	0.97	0.98	0.98	-0.84	-0.71	0.97	1				
*Co*	0.93	0.92	0.92	-0.76	-0.66	0.96	0.95	1			
*Ni*	0.47	0.51	0.46	-0.42	0.00	0.63	0.54	0.65	1		
*Cr*	-0.09	-0.05	-0.09	0.11	0.32	-0.32	-0.26	0.90	0.43	1	
*V*	-0.49	-0.42	-0.48	0.44	0.59	-0.49	-0.41	-0.33	0.13	0.68	1

(**significant at p <0.01, two-tailed).

**Fig 5:**
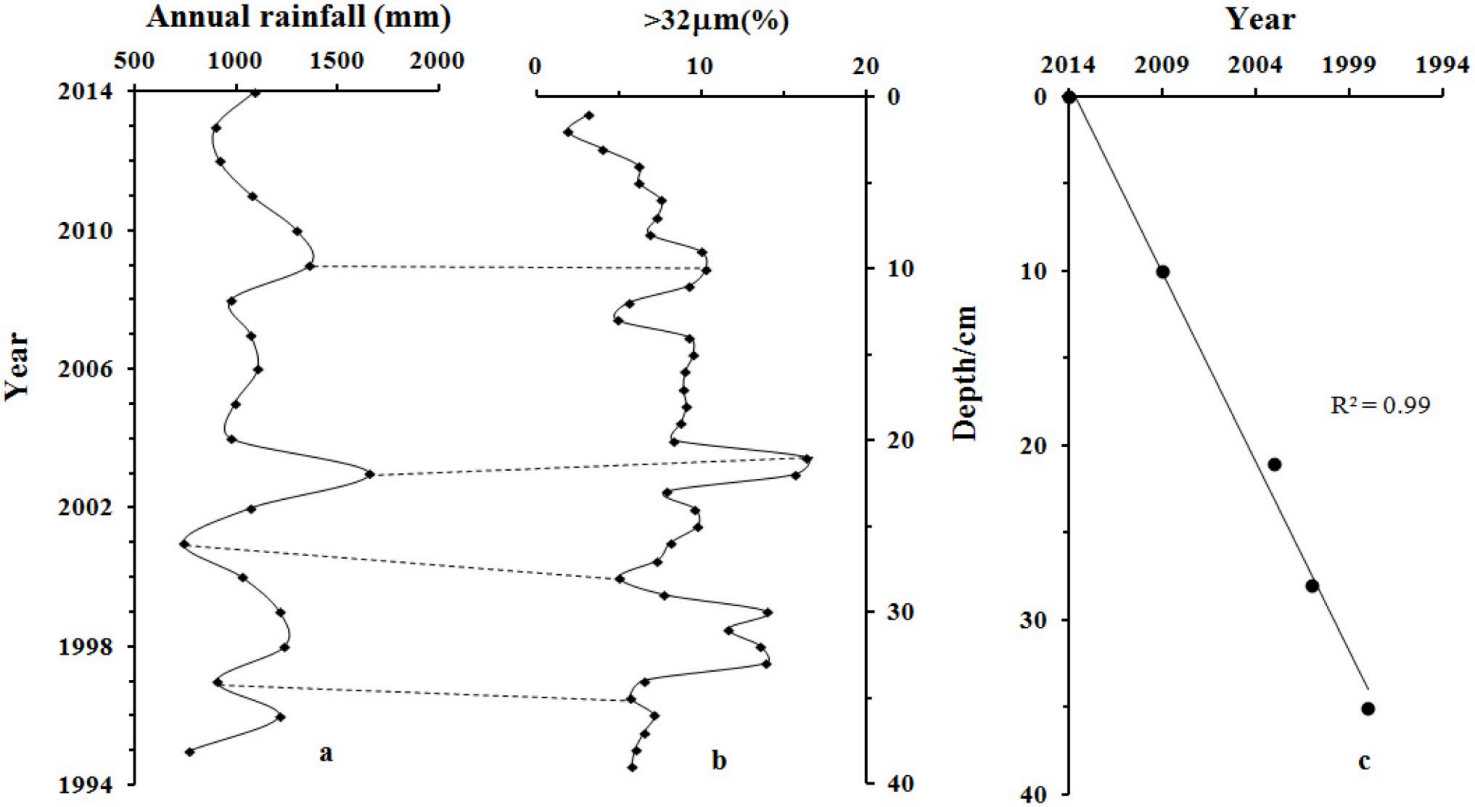
Variations in annual rainfall in Nanjing City (a), >32*μm* particle size concentration (%) of core QH1 (b), and the established age–depth model (c).

The vertical variations of heavy metals (*Zn*, *Pb*, *Co*, *V*, *Cr*, and *Ni*) are also displayed in Fig 5. According to the variation trends, the vertical change of heavy metals were divided into two categories: 1) some heavy metal elements (e.g. *Zn*, *Pb*, and *Co*) exhibited similar trends to those of *χ*, *ARM*, and *SIRM* (Fig 4g–4i). The trends reveal that these heavy metals increased significantly from the bottom to 20 cm before increasing slowly. Table 1 shows that *Zn*, *Pb*, and *Co* had significant positive correlations with *χ*, *ARM*, and *SIRM* (r values of 0.92 0.98). These positive correlations indicate that the magnetic particles may have the same origin, deposition, and migration processes as *Zn*, *Pb*, and *Co*. 2) Some heavy metal elements (e.g. *V*, *Cr*, and Ni) exhibited different trends to those of *χ*, *ARM*, and *SIRM* (Fig 4g–4i); *V* fluctuated from the bottom to the top, *Cr* fluctuated frequently, and *Ni* fluctuated from the bottom to 30 cm, and then from 30 cm to the top. The correlation analysis revealed that *V* was negatively correlated with the magnetic parameters (r values of -0.42 to -0.48), *Ni* was weakly positively correlated with the magnetic parameters (r values of 0.46 0.51), and Cr was not correlated with the magnetic parameters.

## Discussion

### Chronological framework

An accurate chronological framework is key to reconstructing pollution history. As mentioned, no rivers flow into Qianhu Lake; hence, lake water is mainly derived from rainfall and catchment runoff. Runoff, which is caused by rainfall, can erode the topsoil on the surfaces of the surrounding catchment and transport it into the lake basin. Erosion is stronger under heavier rainfall, thus transporting more coarse particles into the lake basin. Therefore, we compared the annual rainfall of Nanjing City and the percentage concentration of coarse particles (represented by >32*μm*) in core QH2. As shown in Fig 5, the variation in the >32*μm* particle size concentration (Fig 5b) corresponded well with the annual rainfall since 1995 (Fig 5a); there was a higher percentage of coarse particles in years with higher rainfall, and vice versa. Therefore, a chronological framework could be obtained, and the bottom age of core QH2 could be calculated to be 1994 using linear extrapolation. The sedimentation rates derived from the age–depth model of core QH1 (Fig 5c) were relatively constant (mean sedimentation rate of 1.8 cm/y), further confirming the validity of the established chronological framework.

### Source of pollutants and pollution history reconstruction

Based on the results of IRM acquisition and backfield curves, *κ*–T curves, and hysteresis loops, it can be concluded that the magnetic minerals of core QH2 were mainly ferrimagnetic minerals such as magnetite and maghemite. Generally, the two main sources of magnetic minerals in lake sediments are those sourced externally and those formed spontaneously. Among them, authigenic minerals are mainly iron sulfides (e.g. pyrite and pyrrhotite), which are usually produced during early reduction and diagenesis processes [43–45]. Previous studies have shown that after magnetic minerals in a watershed enter a lake, they change from a continental oxidation environment to an underwater reduction environment, such that magnetic minerals undergo early diagenetic reduction. The dissolution of iron oxides (e.g. magnetite and maghemite), the formation of iron hydroxides (e.g. goethite) and iron sulfides (e.g. pyrite and pyrrhotite) are representative [46–48]. Core QH2 had a relatively high mean deposition rate of 1.8 cm/y, which may have resulted in a lower degree of diagenesis for the magnetic properties of the sediments [45]. In addition, the results in Figs 3 and 4 show that the main magnetic minerals in the samples were magnetite and maghemite. No signs of iron hydroxide or sulfides were observed in the high-temperature *κ*–T curve. Therefore, we can conclude that the reductive dissolution and transformation of the original magnetic minerals were not significant in the studied cores.

As mentioned above, magnetic minerals are carried from outside the lake sediments. These externally carried magnetic minerals can be divided into natural and anthropogenic magnetic minerals. Owing to the pedogenesis of topsoil, a large number of fine-grained magnetic particles (SP and SD) are generated under natural conditions [32–36]. This results in positive correlations between concentration-related magnetic parameters (*χ*, *ARM*, and *SIRM*) and particle size-related magnetic parameters (*χ*_*fd*_% and *ARM*/*SIRM*). As shown in Fig 5 and Table 1, there were significant negative correlations between the concentration-related magnetic parameters and particle size-related magnetic parameters, implying that the magnetic minerals in the samples did not originate from the pedogenesis of topsoil. Previous studies have shown that the magnetic minerals produced by human activities are mainly coarse particles (multidomain, MD), which have high *χ*, *ARM*, and *SIRM* values but low *χ*_*fd*_% and *χ*_*ARM*_/*SIRM* values [14, 49–52]. Therefore, the change in the magnetic mineral content of core QH2 could relate to human activities.

A review of relevant publications revealed that the pollution history of a lake is often closely linked to local industrial development or urban expansion [28–30, 53]. Industrial activities and urban expansion discharge large amounts of heavy metals and magnetic particles, resulting in positive correlations between magnetic parameters (e.g. *ARM* and *SIRM*) and heavy metal elements (e.g. *Fe*, *Cr*, *Mn*, *Ni*, *Cu*, *Zn*, *Pb*, *Co*, and *Cd*) [49, 54, 55]. However, different heavy metals are associated with different pollution sources; therefore, heavy metals can be used to determine the source of pollutants [54, 56]. As shown in Fig 5 and Table 1, *χ*, *ARM*, and *SIRM* exhibited significant positive correlations with Zn, Pb, and Co, but weak positive correlations with *Ni*, no correlation with *Cr*, and negative correlations with *V*. These results indicate that the magnetic mineral particles and *Zn*, *Pb*, and *Co* had similar sources as well as similar sedimentation migration processes, whereas the magnetic mineral particles and Ni, Cr, and V may have had different sources. Many studies have shown that *Zn*, *Pb*, and *Co* can be produced by both industrial emissions and traffic activities. For example, Zn can be produced from the abrasion of brake linings [57–59]. In addition, Zn is also present in the tires and fuels of automobiles in the form of oxides [60, 61]. Generally, the incomplete combustion of leaded gasoline produces Pb, and Pb also comes from the corrosion of brake linings, engine parts, and car bodies [10, 58, 62]. Moreover, *Co* has a very good effect on improving the adhesion between rubber and metal, and is widely used in automobile tire manufacturing. Therefore, the changes in *χ*, *ARM*, *SIRM*, and heavy metals *Zn*, *Pb*, and *Co* may have an effect on the history of traffic pollution in the study area.

### Reconstruction of traffic-related pollution history

**Fig 6:**
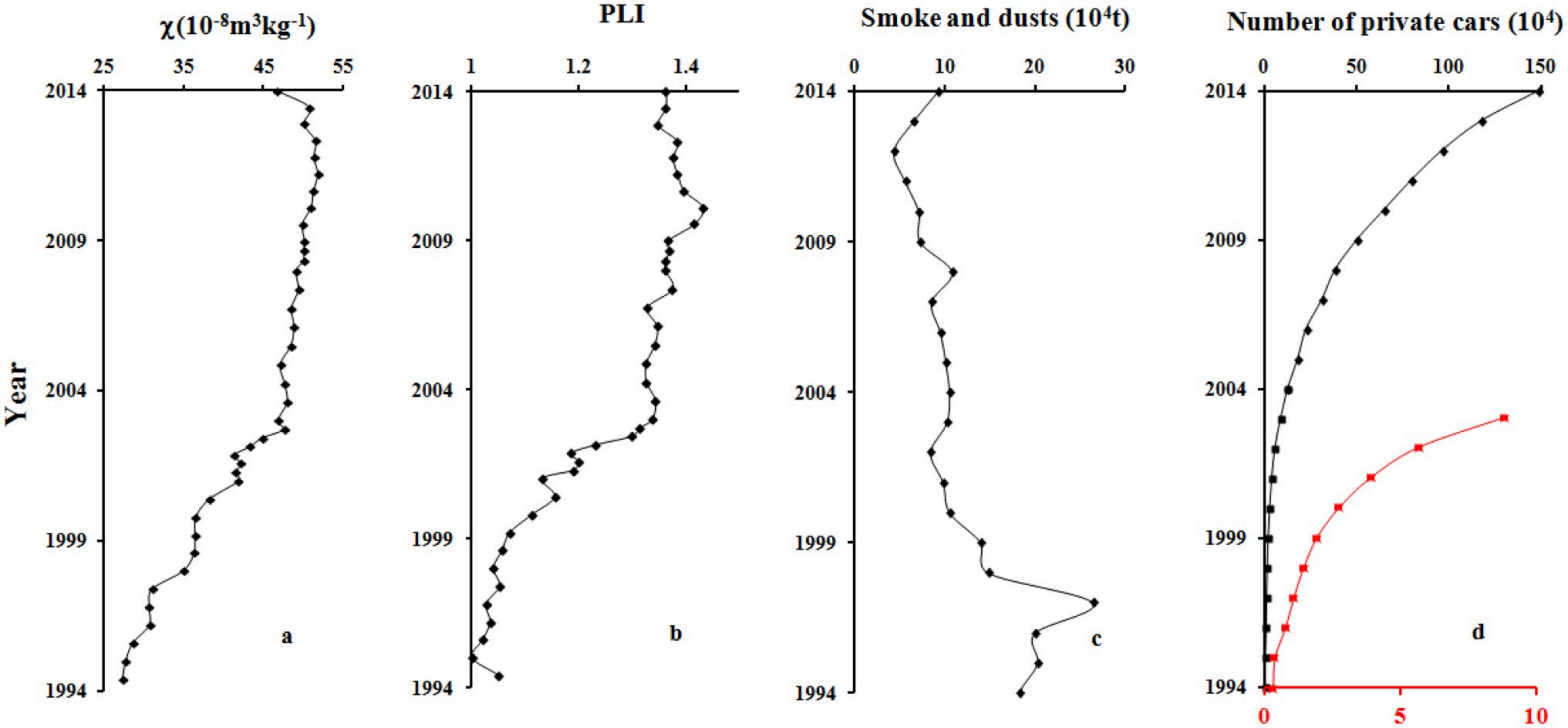
Variations in *χ* (a) and the PLI (b) in core QH1. Variations in annual smoke/dust emissions (c) and the number of private cars in Nanjing City (d) from 1994 to 2014 (the red line indicates the number of private cars from 1994 to 2003).

To further determine the pollution sources, we calculated the pollution load index (PLI) of *Zn*, *Pb*, and *Co* at each depth in the core. This index was calculated as follows:

$$\begin{array}{c} CF_{z}=C_{mz}/C_{bg} \end{array}$$ (1)

$$\begin{array}{c} PLI_{z}=\sqrt[{n}]{\left(CF_{1z}\times CF_{2z}\times \ldots \times CF_{nz}\right)} \end{array}$$ (2)

where *z* is the depth, *CF*_*z*_ is the concentration factor of the respective metal, *C*_*mz*_ is the metal concentration, and *C*_*bg*_ is the mean background concentration of the respective metal ([53]). The lowest value for each metal was selected as the *C*_*bg*_. We then combined the PLI, *χ* statistical data on industrial emissions, and the number of private cars in Nanjing City since 1994, as shown in Fig 6. The variations in *χ* (Fig 6a) and the PLI (Fig 6b) were similar, but different to the variation in industrial emissions in Nanjing City (Fig 6c, represented by annual smoke and dust emissions). The *χ* and PLI values increased rapidly from 1994 to 2003 before steadily increasing from 2003 to 2014, while industrial emissions decreased significantly from 1994 to 2003 and continued to decline from 2003 to 2012, suggesting that the pollution history indicated by *χ* and the PLI was not caused by industrial activities. Fig 6d also shows the number of private cars since 1994, which was similar to the variations in the *χ* and PLI values, especially from 1994 to 2003 (the red line in Fig 6d). They all exhibited significant increases, probably related to the expansion of tourism. There were only a few private cars and tourists before the 1990s; hence, traffic pollution was not serious. With the increase in cars and tourism since the 1990s, an increasing number of tourists chose to travel in their own cars. This generated an increasing number of magnetic particles and heavy metals through exhaust emissions, friction between automobile components, and friction between cars and the road surface, leading to increases in *χ* and the PLI from 1994 to 2003. Although the number of private cars still increased exponentially after 2003, the *χ* and PLI values increased slowly. This could relate to the maximum carrying capacity of the tourist area, whereby the number of cars and tourists nearly approached saturation in 2003. Another reason is technological innovations in automobile manufacturing, such as improvements in exhaust treatment and the complete combustion of fuels, as well as improvements in fuels (e.g. the elimination of leaded gasoline).

## Conclusions

Most existing research has focused on the spatial pattern of traffic pollutants along roads and the factors that influence this pattern. In this study, core sediments with an accurate chronological framework from Qianhu Lake were obtained, and magnetic and chemical parameters were measured in detail. These data were combined with statistical data on industrial emissions and private cars in Nanjing City to reconstruct the history of traffic-related pollution from 1994 to 2014. The results of this study demonstrate the following.

1. Magnetic particles and *Zn*, *Pb*, and *Co* in the studied core sediments were primarily released by traffic activities related to tourism, indicating that their variations in concentration could be used to indicate the pollution history caused by local traffic.
2. Combined with the obtained chronological framework and the variations in the and PLI values, the pollution history caused by traffic in the study area was divided into two stages: 1) from 1994 to 2003, when traffic-related pollution became increasingly serious due to the exponential increase in the number private cars and the prosperity of tourism; and 2) from 2003 to 2014, when traffic-related pollution increased continuously, but at a much slower rate than in stage 1. This slower rate was probably related to the maximum carrying capacity of the tourist area and technological innovations in automobile manufacturing, as well as improvements in fuels. Providing that there are suitable materials for study (e.g. lake sediments), traffic-related pollution history can be accurately reconstructed using magnetic methods, which are both rapid and economical.

## Supporting information

S1 Data (XLS)
